# Strategic Uses of Facebook in Zika Outbreak Communication: Implications for the Crisis and Emergency Risk Communication Model

**DOI:** 10.3390/ijerph15091974

**Published:** 2018-09-10

**Authors:** May O. Lwin, Jiahui Lu, Anita Sheldenkar, Peter J. Schulz

**Affiliations:** 1 Wee Kim Wee School of Communication and Information, Nanyang Technological University, Singapore 637718, Singapore; tmaylwin@ntu.edu.sg (M.O.L.); anitas@ntu.edu.sg (A.S.); 2 Division of Psychology, Nanyang Technological University, Singapore 637332, Singapore; 3 Institute of Communication and Health, Università Della Svizzera Italiana, 6900 Lugano, Switzerland; peter.schulz@usi.ch

**Keywords:** Zika, CERC, Facebook, social media, outbreak communication, crisis communication, public health

## Abstract

While social media has been increasingly used for communication of infectious disease outbreaks, little is known about how social media can improve strategic communication across various stages of the health crisis. The Crisis and Emergency Risk Communication Model (Reynolds & Seeger, 2005; CERC) outlines strategies across different crisis phases and can guide crisis communication on social media. This research therefore investigates how social media can be utilized to implement and adapt the CERC model, by examining the strategic uses of Facebook in communicating the recent Zika epidemic by health authorities in Singapore. Zika-related Facebook posts of three main Singapore health agencies published within the one year period from January 2016 to December 2016 were thematically analysed. Results suggest that Facebook was used to communicate the crisis strategically, which supported and added to the CERC model. Novel uses of Facebook for outbreak communication were demonstrated, including promoting public common responsibility for disease prevention and expressing regards to the public for cooperation. Results also suggested that preparedness messages might be the most effective, as they produced a great level of public engagement. The adaptability of the CERC model in social media contexts to improve crisis communication is discussed.

## 1. Introduction

With the proliferation of the Internet and mobile phones, social media is playing an ever-increasing role in crisis communication during infectious disease outbreaks. Due to its conversional and transparent characteristics, social media allows for health authorities to post real-time information as a crisis unfolds, as well as quickly reach a large number of people at a low cost [1,2]. In addition, as social media allows two-way communications between health authorities and the public, health authorities can quickly address public concerns and reduce public panic during the crisis [3]. The benefit of social media can be highlighted in health communication crises, such as an infectious disease pandemic outbreak, as uncertainties and high perceived risks from the public are expected and require constant and strategic communication with the public on the rapidly changing situation [4]. Thus, social media could likely improve strategic communication by linking the public with real-time updates of the crisis and information on how the health organizations are functioning. However, little is known about how social media can improve strategic outbreak communication.

The Crisis and Emergency Risk Communication Model (CERC) has been widely adopted for strategic communication in risk and crisis situations. CERC is a communication model that outlines the five common stages of the risk and crisis lifecycle, “from risk, to eruption, to clean-up and recovery, and on into evaluation [5] (p. 51)”. It provides strategic practices during each stage to the best communicate risks and reduce uncertainty, and thus can potentially fill the research gap.

This research aims to analyse how social media may be utilized to implement and adapt the CERC model. The recent Zika epidemic in Singapore was used as a case study because it provides a valuable opportunity to investigate the strategic use of social media in communicating an infectious disease outbreak. Singapore is a technology-rich country and has been honoured by the World Health Organization (WHO) as a role model in managing Zika outbreak [6]. Leading Singapore health authorities have utilized social media for the timely management of the outbreak and the public has actively engaged in outbreak messaging [7]. Therefore, examining strategic communication while using social media by health authorities in the context of Singapore Zika outbreak should offer valuable insights into the CERC model.

### 1.1. Outbreak Communication on Social Media

Outbreak communication is a type of crisis communication. Unlike other organizational crisis, uncertainties and high perceived risks from the public are expected in pandemic and epidemic situations [4]. This is especially true in the case of Zika outbreak because of its ease of transmission and long-term risks to new-born babies. One major goal of outbreak communication is therefore to accurately portray risk and disseminate information, while at the same time, avoiding projecting overconfidence or inducing panic among the public [8,9].

With a proliferation of users, health authorities are increasingly utilising social media for outbreak communication. Social media, such as websites or online platforms that allow for users to communicate or follow each other, provide newfound mechanisms for receiving and contributing information, often in real-time [10]. Rather than simply supporting exchange of messages among a small group of friends, social media support rapid dissemination of information to diverse users because information can be shared and re-shared through users’ social networks [1,2]. Social media also encourages efficient social connections while promoting fruitful conversation [11,12]. People can express their opinions by commenting on the information. Therefore, social media allows for health authorities to spread valuable information to and obtain timely reactions from a large audience.

Empirical research has demonstrated that social media could be used for communicating outbreak-related updates and information by health authorities during an outbreak to improve awareness and response. A number of studies have investigated the use of social media during an infectious disease epidemic [4,7,13,14,15]. For example, government health organizations, such as the United States (US) Centers for Disease Control and Prevention (CDC) and the World Health Organization (WHO), have used social media platforms such as Twitter and Facebook to communicate information about Ebola-related news, crisis responses, prevention, and potential danger during the Ebola outbreak [14]. Vijaykumar and colleagues [7] found that the two major government health agencies in Singapore, Ministry of Health (MOH) and National Environmental Agency (NEA), used Facebook to communicate the 2016 Zika outbreak by posting information on the situation and prevention measures.

Though less common, social media can also be used to reduce public panic and build trust from the public during a disease pandemic. For example, during the 2009 H1N1 epidemic, government organizations, such as WHO and CDC, not only informed the public about H1N1, but also expressed sympathy for those affected and reassured the public that actions had been planned to solve or prevent the crisis [15]. Within a few months of the first imported US Ebola case, local health departments in US posted information that directly aimed at dismissing fear toward Ebola and promoted events to answer public concerns [16].

Although the above research provides evidence of social media use for outbreak communication, it extensively focused on the phase when the outbreak is present [17]. Less is known about how social media should be strategically used by health authorities to disseminate various types of messages across different phases of a crisis. Theories have suggested that public audiences have various information needs across different stages of a crisis [18]. In the pre-crisis stage, the public may tend to seek information concerning the nature of the potential crisis itself. Whereas, in the outbreak phase, the public may seek out information about details of the situations and attend to behavioural recommendations that can help protect oneself from the crises [5,19]. This suggests that effective crisis communication requires strategic messaging that meets audiences’ information needs across different phases of a crisis.

The limited research on social media use across different time periods of a disease outbreak showed mixed results on whether social media was utilized strategically by authorities. For example, Wong et al. [16] found that trends in Ebola tweets by local health departments were aligned with major milestones that took place throughout the 2014 West Africa Ebola outbreak. Vijaykumar et al. [7] also found that the health authorities in Singapore posted more Facebook updates when there was a locally transmitted Zika outbreak. In contrast, Liu & Kim [20] found that, during the H1N1 outbreak, government health organizations did not actively attempt to address the emotional needs of the public around the crisis’ milestones. Specifically, outbreak responses on Twitter and Facebook by government organizations did not differ in frequency of information in addressing public alarm or providing sympathy before and after the declaration of pandemic by the WHO and the vaccine development announcement. Thus, how social media can be strategically used to disseminate various messages across different outbreak phases remains unclear.

Furthermore, given that the public show varied interests in different information, messages may have varying influence or value to the public, which will be reflected in public reactions to the social media posts [14,21]. Social media, such as Facebook, offers several ways for users to react and respond to messages, such as “like”, “share”, and “comment”. These responses give an indication of the collective perceptions towards the messages. For example, liking can indicate public interest in the posts and signal the value of the message [22,23], because likes are often considered as a proxy of message impact and effectiveness [24,25]. Sharing and commenting can indicate the relevance of the topic [23]. While sharing indicates the intention of users to disseminate the information to their social networks, the comments can convey user attitudes and opinions towards the message [26], influencing other audiences’ evaluations on the quality and credibility of the original message [27]. Therefore, investigating public reactions to government messaging along the outbreak timeline should have significant implications on evaluating the effectiveness of health authorities’ communication strategies.

### 1.2. The CERC Model

Theoretical models have incorporated social media into health and crisis communication [28,29]. Veil et al. [29] summarized the top ten ways to use social media in risk and crisis communication, such as communicating with honesty and providing messages of self-efficacy. While most of these theoretical efforts identify the best practices of social media uses in crisis communication, they do not specify in which phase of the crisis the practices should be utilized by health authorities. Houston et al. [28] developed a functional framework by identifying social media users, such as communities, governments, and individuals; and, describing correspondent practices in different phases of a crisis. However, this framework did not specifically focus on strategic communication of health authorities. Therefore, there is an urgent need for a model that guides health authorities’ strategic communication across various crisis phases on social media.

The Crisis and Emergency Risk Communication (CERC) model may potentially be able to fill this gap within the communication framework literature. It is a stage model that was developed by the US Centers for Disease Control and Prevention and it has been widely adopted to manage risk communication during public health crises [5]. Table 1 shows the CERC model’s five common stages, along with each stage’s communication characteristics and the corresponding good practices, as adapted from [5].

The pre-crisis phase is the first stage of the model and it is characterized by risk messages, warnings, and preparations. At this stage, communicators should provide educated information and warnings of the potential risk, identify responders and spokespersons, and encourage self-protection actions. The second stage is the initial event when the crisis occurs. During this phase, communicators should reduce uncertainty by providing timely updates of the crisis and messages of self-efficacy and personal response activities. They should also reassure the public about interventions that have been employed to improve the situation. Stage three is the maintenance stage in which communicators should continue with reassurance, uncertainty reduction, and encourage self-efficacy. Stage four is the resolution phase, involving updates of on-going resolutions, discussions about causes, and new understandings of risk. The final stage is the evaluation phase, characterized by evaluating response and communication effectiveness and reaching consensus on lesson learned.

The CERC model helps to “prevent further illness, injury, or death; restore or maintain calm; and engender confidence in the operational response” [30] (p. 249). By emphasizing various communication needs of the audience at various points of a crisis event, the model outlines specific kinds of communication strategies that should be used at various stages in the ongoing development of a crisis. This allows for health communications to be strategic, broad based, responsive, and highly contingent [5,31].

However, existing literature on the CERC model has yet to be fully tested and adapted CERC in social media contexts during a disease epidemic. Little research has been done on how well government health authorities follow the CERC model on social media during disease outbreaks. This is important to explore, because an infectious disease epidemic may typically follow the CERC pattern more than other types of crises [5], and its investigation can thus provide strong evidence and implications to the CERC model. In addition, though the CERC model suggests potentially demanding strategies of various stages of a crisis, examinations of strategy effectiveness is lacking, especially in social media contexts.

Therefore, in this research, we aim to fill the research gap by analysing how social media may be utilized to implement and adapt the CERC model. We aim to demonstrate strategical uses of Facebook for communicating the Zika epidemic in Singapore. Singapore is world’s second best country for digital connectivity. Approximately 91% of its citizens are using smart phones and 82% are active internet users. Health issues have been intensively discussed on social media, such as Facebook. For example, as of 2016, official Facebook pages of government health agencies have more than 100,000 followers and received more than 100,000 likes from the public [7]. In addition, Singapore has been honoured as a role model in managing Zika crisis by WHO. Therefore, the findings are expected to provide insights on an adapted CERC model for social media.

### 1.3. The Zika Epidemic in Singapore

Zika, the mosquito-borne infectious disease that has triggered alerts around the globe, attracted nationwide attention in Singapore during the 2016 outbreak. The Zika disease is easily transmitted as 80% of the infected cases display no symptoms. The disease also raises long-term risks for pregnant women and their newborns who are more likely to be diagnosed with microcephaly and developmental issues. As a result, public concern about the Zika outbreak quickly grew, particularly among pregnant women [32].

In January 2016, the Singapore government added the Zika virus to the List of Notifiable Infectious Diseases under the Infectious Diseases Act and issued a circular to doctors to heighten awareness of the virus. After the WHO’s declaration of Zika as a global public health emergency in February 2016, the government announced more measures to strengthen the preparations against the virus. On 13 May 2016, the first imported case of Zika was confirmed and on 27 August 2016, the first locally transmitted case was verified. The following day, the government confirmed the localised spread of Zika, signifying the Zika outbreak in Singapore. From 27 August to 19 September 2016, 381 confirmed cases were reported. On 19 September 2016, the first Zika cluster was announced to be closed and the weekly reported case of Zika declined to a low level until the end of the year.

The outbreak triggered a large scale conversation about Zika on social media. Facebook is one of the major platforms for the discourse of Zika in Singapore, as it is the second most active social media channel with 72% of Singapore’s 5.7 million residents being active users. Even government agencies, such as Minister of Health (MOH) and National Environment Agency (NEA), used Facebook to engage and inform the public about the Zika outbreak [7]. Therefore, the Zika epidemic in Singapore provides a good opportunity to investigate the government communication of a disease outbreak on social media.

## 2. Method

To understand how Zika was strategically communicated by the government across various stages of the epidemic, an in-depth content analysis was conducted on Facebook posts for the three key government agencies, namely National Environment Agency (NEA), Ministry of Health (MOH), and Health Promotion Board (HPB). The three government authorities selected are responsible for preventing and communicating large-scale health crises and were therefore considered the main government sources of information. Public responses to the original government posts, such as likes, shares, and comments were also analysed to understand the effectiveness of the communication. In order to compare social media data to the official Zika figures, the data were analysed according to epidemiological weeks (i.e., epi-week).

To fully capture the government communication of Zika outbreak in 2016 and maximize our data source, the time of the first Zika case (13 May 2016) was considered as the midpoint, and extended the investigation to the whole year of 2016. This can ensure that our investigation covers the various stages of the Zika epidemic in Singapore. As a result, government posts containing the keyword “Zika” from 1 January 2016 (i.e., epi-week 52, 2015) to 31 December 2016 (i.e., epi-week 52, 2016) were collected. Public responses to the original government posts, such as likes, shares, and comments were also collected. For reference and comparative purposes, we downloaded official data from the MOH on reported confirmed Zika cases in Singapore.

To examine strategic communication of the three authorities, the study used a combination of analytic and grounded factors for content analysis. For the analytic factors, six major themes were adapted from the CERC: (1) risk messages: posts containing information of disease mechanisms and symptoms; (2) warnings: posts highlighting risk factors and dangers associated with Zika; (3) preparations: posts mentioning first responders and providing response recommendations; (4) uncertainty reduction: posts containing information of case reports, local areas, and information sources; (5) efficacy: posts mentioning specific personal prevention measures and highlighting common responsibility for disease prevention; and, (6) reassurance: posts that calmed the public with mentions of government interventions, and expressed thanks and regards to the public efforts (Reynolds & Seeger, 2005). Then, all the authors read the posts independently, and identified the grounded emerging topics and their defining keywords for each major theme from the text data through an open coding process. Strauss & Corbin [33] describes open coding as a process of “naming and categorizing phenomena through close examination of the data”. The team met to discuss disagreements and agreed on the final grounded topics and keywords, from which the codebook was generated (Table 2). The codebook was then used methodically to categorise the posts. Through the open coding process, government was found to regularly mentioned dengue in their Zika posts. Given that dengue shares the same mosquito vector to Zika, an additional major theme for “dengue” was added. The theme of dengue includes not only mentions of the disease itself, but also the new technology that uses Wolbachia, which is a bacteria that can help to reduce populations of the Aedes aegypti mosquito that carries the Zika and dengue viruses [34].

Furthermore, to capture the content of comments to government posts, keyword extraction analysis was conducted, using the “udpipe” R package for natural language processing. Keyword collocation method was used to detect common expressions up to 4 g. Extracted expressions were plotted into word clouds.

The investigation periods were categorised into three phases according to the CERC model. The pre-outbreak phase was operationalized as from 1 January 2016 (epi-week 52, 2015) to 20 August 2016 (epi-week 33, 2016); the outbreak phase was from 21 August 2016 (epi-week 34, 2016) to 24 September 2016 (epi-week 38, 2016), which is corresponding to the CERC initial event phase; the post-outbreak phase was from 25 September (epi-week 39, 2016) to 31 December 2016 (epi-week 52, 2016), which incorporates CERC phases of maintenance, resolution, and evaluation. The three phases were categorized as above because the outbreak began on 27 August 2016 (epi-week 34, 2016) with the first confirmed locally transmitted case of Zika, and ended on 19 September 2016 when the first local Zika cluster was announced to be closed by the government (epi-week 39, 2016).

## 3. Results

Overall, the extraction totalled 72 Zika-related posts, with 33 posts from NEA, 37 posts from MOH, and two posts from HPB. They produced a total of 2011 likes, 1185 shares, and 236 comments. In addition, they produced a median of 17.5 likes, zero shares, and two comments. Results for government posts are first presented and then public reactions to these original posts.

### 3.1. Government Posts

**Figure 1:**
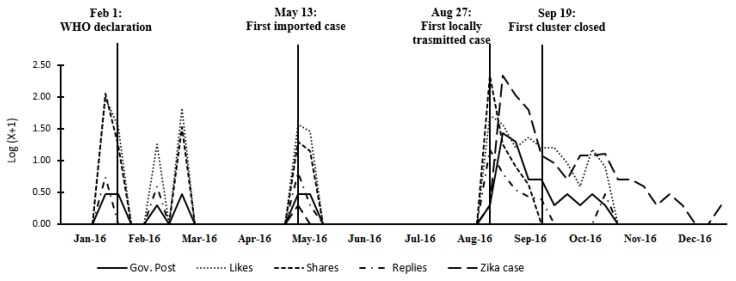
Facebook Zika communications in relation to confirmed Zika cases and key Zika activities in Singapore.

The confirmed reported Zika cases were plotted to provide a reference point in relation to the Facebook government communication of the disease. As seen in Figure 1, health authorities began to post Zika information when the disease raised national and international concerns in late January 2016, signified by the addition of Zika into the List of Notifiable Infectious Diseases in Singapore and the WHO declaration of the disease as a public health emergency of international concern. They then provided updated information when the first imported case was confirmed on 13 May. Overall, 15.3% of posts (*N* = 11) were updated at the pre-crisis phase. On 27 August, the government confirmed the first locally-transmitted Zika case and then declared the disease outbreak. Zika posts were consistently updated until the number of new cases fell to a significantly lower level, accounting for 75.0% of all posts (*N* = 54). Then, health authorities provided follow-up Zika information until the end of the year, updating 9.7% of all posts (*N* = 7). This suggests that health authorities strategically updated timely posts when there were important developments in Zika activity in Singapore.

Table 3 shows results of thematic analysis of posts. Of the 72 posts, 90.3% of all posts contained information for preparations and 87.5% contained uncertainty reduction information. 80.6% of all posts contained risk messaging, followed by 76.4% of all posts contained reassuring information and 72.2% on warning messages. Efficacy information constituted 69.4% of all posts and 33.3% of the posts contained information relating to dengue.

For risk message posts, while 62.5% of posts mentioned the disease mechanisms, only 37.5% provided information on disease symptoms. Within the warning theme, 68.1% of posts mentioned risks that are associated with Zika infection and 26.4% of posts directly emphasized the danger of the Zika disease spread. For messages about preparedness, 73.6% of posts mentioned first responders and 55.6% of posts provided recommendations to reduce harm. Within the uncertainty reduction theme, posts that provided resources, reported cases, and mentioned specific local areas were all prevalent topics, accounting for around or above 70% of all posts. For the efficacy theme, 62.5% of posts emphasized public collaboration to prevent Zika. Approximately half of all posts (47.2%) mentioned specific actions for personal protection from the disease. The majority of reassurance themed posts tried to assure the public that the government has conducted or will promote efforts to monitor and improve the situation (72.2%), with over half of all posts mentioning specific government interventions (59.7%). Finally, 33.3% of all posts linked Zika with dengue and 2.8% of posts mentioned Wolbachia, a new technology for controlling mosquito vectors.

In order to understand how health authorities strategically use Facebook for Zika communication, cross tabulation analyses were conducted by examining relationships between post themes by crisis phases (Table 3). Posts about risk messages, preparations, and uncertainty reduction share the same pattern across phases. These posts accounted for a high proportion of all posts in the pre-crisis (risk messages, 91%; preparations, 82%; uncertainty reduction, 91%) and the initial event phase (risk messages, 83%; preparations, 98%; uncertainty reduction, 93%), but witnessed a significant drop in the maintenance phase (risk messages, 43%, *χ*^2^ = 7.37, *p* = 0.025; preparations, 43%, *χ*^2^ = 22.64, *p* < 0.001; uncertainty reduction, 43%, *χ*^2^ = 14.15, *p* < 0.001). The warning posts dropped in frequency across the three phases, from 91% in the pre-crisis phase to 29% in the maintenance phase (*χ*^2^ = 8.66, *p* = 0.013). The percentage of reassurance posts peak in the initial event phase (84%), when compared with the pre-crisis phase (64%) and the maintenance phase (29%) (*χ*^2^ = 12.18, *p* = 0.002). Conversely, the frequency of efficacy posts was the lowest in the initial phase (65%), as compared with the pre-crisis (82%) and the maintenance (100%) phase, though the differences were only marginally significant (*χ*^2^ = 4.94, *p* = 0.084). Finally, although the dengue posts were most frequent in the maintenance phase, the differences were not significant among phases (*χ*^2^ = 2.17, *p* = 0.34).

For subcategory themes, posts about risk factors (91%) and dangers (64%) of the disease were mentioned the most in the pre-crisis phase, and significantly fell in the later phases (risk factors, *χ*^2^ = 7.67, *p* = 0.022; dangers, *χ*^2^ = 10.85, *p* = 0.004). Posts mentioning first responders accounted for a high proportion of posts in the pre-crisis (64%) and initial event (81%) phase, and the proportion significantly decreased in the maintenance phase (29%, *χ*^2^ = 9.60, *p* = 0.008). Posts providing case report and information resources had a high percentage of posts in the pre-crisis (case report, 73%; information resources, 73%) and initial event (case report, 78%; information resources, 83%) phase, but witnessed a drop in the maintenance phase (case report, 29%, *χ*^2^ = 7.48, *p* = 0.024; information resources, 43%, *χ*^2^ = 6.07, *p* = 0.048). In addition, posts mentioning local area and calming posts peaked in the initial event phase (78%, *χ*^2^ = 9.95, *p* = 0.007, and 83%, *χ*^2^ = 13.90, *p* < 0.001, respectively).

### 3.2. Public Responses to the Government Posts

Figure 1 also plots the averaged public responses across time. The public responses did not accurately align with the Zika milestone activities. Rather, the public reacted to government messaging the most for the first few Zika posts after each interval when Zika information was not updated.

Since social media responses are not normally distributed, the median rather than the mean was adopted for statistical comparison. The Kruskal Wallis test was used to check for differences in engagement across outbreak phases. Mann Whitney U tests were used to examine the engagement differences between posts mentioning versus not mentioning different topics. Table 4 shows the median of public responses across different stages of the outbreak and themes. Table 5 shows public median responses in posts with versus. without topics across crisis stages.

***Like.*** As shown in Table 4, For all posts, the public liked messages the most before the outbreak, and the frequency of likes decreased as the outbreak unfolded (Median = 43, *χ*^2^ = 15.77, *p* < 0.001). Looking at the different themes of posts, the public were found to like posts encouraging self-efficacy the most (Median = 22). Mann Whitney U tests showed that the frequency of likes was significantly higher for posts that encouraged the self-efficacy than posts that did not (*Z* = 2.13, *p* = 0.033). Further analysis (Table 5) showed that posts in this theme received more likes during the outbreak phase (*Z* = 2.72, *p* = 0.007).

***Share.*** For all posts, the public also shared messages the most before the outbreak (Median = 26, *χ*^2^ = 8.71, *p* = 0.013), while the number of shared messages decreased in the post-outbreak phase. Examining the themes of the messages, the public shared the most reassuring posts (Median = 3), followed by risk messages (Median = 2.5). Mann Whitney U tests revealed that the number of shares was significant higher for posts that provided risk messages (*Z* = 3.35, *p* = 0.001), uncertainty reduction (*Z* = 2.89, *p* = 0.004), and reassurance (*Z* = 2.96, *p* = 0.003) than posts that did not. In contrast, posts mentioning dengue received fewer shares (*Z* = −2.99, *p* = 0.003). Concerning posts in different outbreak phases, Table 5 further shows that posts containing risk messages (*Z* = 2.60, *p* = 0.009), uncertainty reduction (*Z* = 1.96, *p* = 0.05), and reassurance (*Z* = 2.90, *p* = 0.004), were shared more in the outbreak phase.

***Comment.*** For all posts, unlike the pattern of like and share, the public commented the most during the outbreak phase, *χ*^2^ = 10.71, *p* = 0.005. Looking at the different themes of posts, the public produced a median of two comments for all topics of posts, except that dengue posts received a median of one comments Nevertheless, Mann Whitney U tests showed that comment frequency was significant higher for posts that contained risk messages (*Z* = 1.96, *p* = 0.050), preparations (*Z* = 2.29, *p* = 0.022), uncertainty reduction (*Z* = 3.22, *p* = 0.001), and reassurance (*Z* = 3.20, *p* = 0.001) than posts that did not. Table 3 further shows that, during the outbreak phase, posts containing uncertainty reduction (*Z* = 2.55, *p* = 0.011) and reassurance (*Z* = 2.10, *p* = 0.036) received more comments.

**Figure 2:**
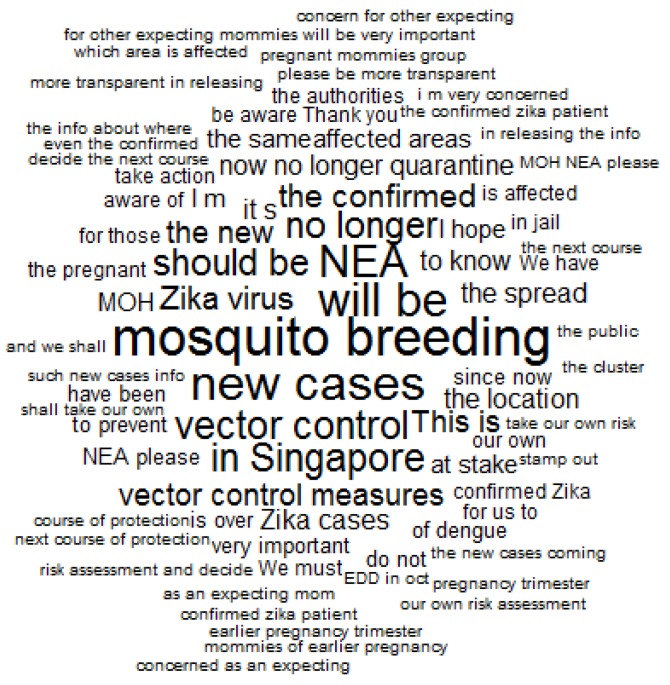
A word cloud for keywords in comments by publics (*N* = 230). Expressions with a frequency higher than 5 were plotted. Larger word size indicates higher use frequency.

The content of the comments were further examined to understand public opinions towards the government posts. As shown in Figure 2, public comments related to different concerns of the Zika outbreak. First, the public expressed concerns with vector control measures for mosquito breeding. They used phases, such as “vector control”, “vector control measures”, “to prevent”, “own risk assessments”, and “next course of protection”. Second, the public expressed their concerns on the spread of Zika cases and their locations. They mentioned phases, such as “new cases”, “confirmed cases”, “affected areas”, “the location”, and “no longer quarantine”. Third, they were also worried about Zika risks on pregnancy. They expressed phases, such as “pregnant mommies group”, “as an expecting mom”, “mommies of earlier pregnancy”, and “earlier pregnancy trimester”. Fourth, the public also submitted their requests to the government, while using expression, such as “NEA please”, “MOH NEA please”, and “more transparent in releasing the info”.

**Figure 3:**
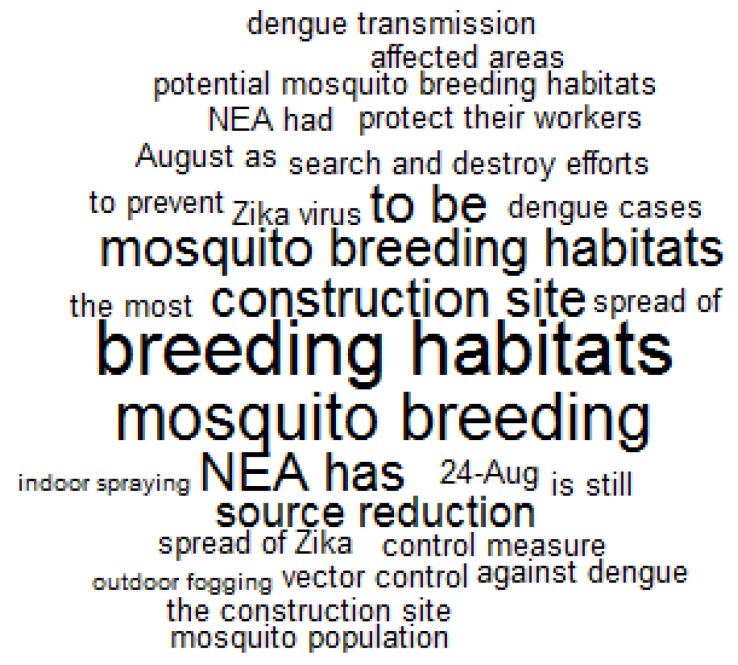
A word cloud for keywords in comments by governments agencies (*N* = 6). Expressions with a frequency higher than 3 were plotted. Larger word size indicates higher use frequency.

Figure 3 indicates that government agencies commented on original posts to reassure the public with efforts that they had taken. For example, the government mentioned “mosquito breeding habitats”, “construction sites”, “control measures”, and “vector control”. They used expressions, such as “NEA has” and “NEA had” to emphasise their “search and destroy efforts” by “indoor spaying” and “outdoor fogging”.

## 4. Discussion

Effective outbreak communication requires strategic messaging across different outbreak phases. As government agencies are increasingly using social media platforms to communicate disease outbreak, it is important to adapt existing models in a social media context to guide their strategic communication. This study demonstrates an early attempt by health authorities to deploy social media as a tool for strategic outbreak communication. It uncovered key messaging strategies that are utilized by health authorities and their potential value and effectiveness, as indicated by the public responses.

First of all, Facebook was used as real-time communication tool as Facebook communication of the Zika disease clearly mirrored the physical disease spread. Specifically, health authorities posted updates the most within the first two weeks after the outbreak was declared. This likely matched the need for information of the public, as audiences would increase social media use for up-to-date information during the crisis [35,36]. In addition, it is noteworthy that health authorities did not update Zika information after announcement of the first imported case in May until that of the outbreak on August. Rather than neglecting Zika, Vijaykumar et al. [7] suggested that this may be because the authorities prioritized information about the generic mosquito-borne diseases over those two months.

Facebook was also used strategically at different outbreak phases by health authorities, with most strategies clearly corresponding to the CERC model. In the pre-outbreak phase, health authorities tended to provide risk messages, including disease mechanisms and symptoms, and warnings, such as potential dangers and risk factors that are associated with the Zika disease. They identified first responders and provided recommendations for public preparedness. In the outbreak phase when the Zika outbreak began, health authorities reduced situation uncertainty and reassured the public. Specifically, health authorities tended towards updating on the disease case reports, and warning citizens in specific locations where Zika had been detected. They also posted calming messages such as information about government interventions and provided further information sources. In the post-outbreak phase, during which the disease has been controlled, authorities again emphasized self-efficacy by highlighting common responsibilities and encouraging personal prevention measures. This suggests that social media can be used to implement the CERC model for strategical outbreak communication.

The findings showed that messages that raised public awareness of the disease received the most public responses. The findings showed that the public liked and shared the most in the pre-outbreak phase. They also reacted the most to the first few Zika posts after each interval when the Zika information was not updated. These patterns corresponded to adaptation level theory of risk stimuli [37,38] and novelty of news value [39] that people only show strong reactions to messages that are initially unfamiliar and against the general routine of people.

The findings also demonstrated that different topics of messages received different levels of responses from the public, indicating their value and relevance to the disease outbreak. The public liked posts the most that encouraged self-efficacy. This suggests that these themed posts were valuable in providing individuals ways to protect themselves from the disease. Also, the public tended to share posts about risk messages, uncertainty reduction, and reassurance. This is likely because those messages can promote their understanding, while at the same time, reduce anxiety towards the Zika disease, and thus are important to be shared among social networks. Furthermore, the public also commented the most on similar messages such as uncertainty reduction and reassurance. This indicates that those messages are extremely relevant to their experiences during the Zika epidemic. Further content analysis shows the public expressed concerns and requests via comments. The above findings support the assertion from the social amplification of risks framework [40] (SARF) that audiences are communication stations that can contribute to increasing information reach and engaging others [41].

More importantly, different topics of messages had varying values as the outbreak unfold, implying the public’s changing psychological needs across the Zika epidemic. In the pre-outbreak stage, the public showed high interest in all Zika messages except those related to dengue. This may be because pre-outbreak Zika posts mentioning dengue in the samples were largely related to the dengue control, but not Zika disease per se, and thus they might not be considered as relevant. During the outbreak, they attended to more relevant messages than others, such as self-efficacy, uncertainty reduction, and reassurance, indicating their needs for preventing oneself from the disease. In the post-outbreak stage, the overall public responsiveness to Zika messaging reduced. This suggests that the public no longer considered the outbreak as a threat at this stage and the information became irrelevant.

### 4.1. CERC in Social Media Contexts

The findings not only demonstrate how social media can be used to implement the CERC model, but also help to adapt the CERC model in social media contexts (Table 6). First, the current study is one of the few endeavours to directly operationalize the CERC model for investigating strategical outbreak communication in social media contexts. In terms of operationalizing outbreak phases, the researchers believe that one of the reasons scholars focused exclusively on the outbreak phase in previous research is that various phases in the CERC model have not been adequately defined and operationalized in the disease epidemic context. An epidemic of an infectious disease may last weeks to months, and the boundaries between stages are less clear than other crises, such as extreme weathers [42,43]. In this study, the model stages were adapted and operationalized into pre-outbreak, outbreak, and post-outbreak phase. This adaptation aligns with previous theoretical effort of Houston et al. [28]. In terms of the operationalization of strategies, six major themes from the CERC model were focused on, and subcategories and new themes emerging from a post-review procedure were considered. The researchers realized that some subcategories can be categorized into different major themes. For example, this study considered personal prevention measures as a subcategory of efficacy [31]. However, it can be also considered as a subcategory of public preparedness in the pre-crisis phase [5]. Therefore, as one of the first attempts to operationalize phases and strategies in the CERC model, further refinement is necessary.

Second, in regards to communication strategies that were adopted during the Zika outbreak in Singapore, the findings provide interesting suggestions for adapting some of CERC response recommendations on various stages in social media contexts. Specifically, the CERC model suggests that risk messages, warnings, and preparations should be the three characteristics in the pre-outbreak phase because the public would tend to seek information related to the nature of the risk itself in this stage. However, the results showed that uncertainty reduction and efficacy were also characterized in pre-outbreak messages on Facebook. Even more, the public showed interest in all Zika-related messages when their awareness of the disease was raised. This suggests that pre-outbreak messages on social media should include all the necessary information to update and educate the public about the risk of the potential crises, while providing reassurance and self-efficacy. This can be important to tackle barriers in pre-crisis communication such as high level of uncertainty and negative emotions during risky situations [44].

CERC also suggests that health communicators should constantly reduce situational certainty and reassure the public during the outbreak stage. Indeed, the findings on government posts provide evidence on the model that government agencies frequently updated on the disease case reports and calmed the public with information about ongoing government interventions. Seemly contradictory, the results about public reactions showed that the public generally engaged much less in the outbreak messages than they did in the pre-outbreak phase. This suggests that the information during the outbreak phase was less informative. Nevertheless, this cannot lead to a conclusion that constantly posting messages during the outbreak phase is unnecessary. Rather, the findings that the public replied more the outbreak messages suggests that the messages were relevant and they could elicit valuable discussions between the public and the government.

Third, the findings also suggest that there are a number of approaches that were designed and driven by social media, which are currently not part of the CERC. For example, health authorities often emphasized the publics’ common responsibilities for disease prevention. They also expressed thanks and regards, though infrequently, for co-operation from the public throughout the outbreak phases. These approaches are different from CERC’s strategies, such as cooperating with agencies, organizations, and groups in the pre-crisis phase, and discussing issues regarding blame and responsibility in the resolution phase [5]. These new approaches are two-way communications in nature that are likely emerge, because social media tend to promote discussion between communicators and audiences. In addition, health authorities sometimes educated the public about Wolbachia, the new technology for mosquito control. This suggests that social media can broaden the scope of health discussions [10,45], no longer focusing exclusively on the current disease situations but also healthcare education.

In sum, the current research demonstrated an adapted CERC model within social media, specifically for disease outbreak contexts (See Table 6). Preparedness messages should include all crisis-relevant information, as they may be the most effective on educating and informing potential outbreaks to the public. Continued and ongoing practices for reducing uncertainty and promoting self-efficacy is needed to reduce the emotional turmoil and elicit discussions between the publics and the governments during the outbreak. Novel uses of social media for outbreak communication are identified in social media contexts, including promoting public common responsibility for disease prevention and expressing regards to the public for cooperation.

### 4.2. Limitation and Future Directions

There are several limitations and future directions to the study. First, while the study focused on Facebook, future research efforts should consider studying the same topic on Twitter, Instagram, and similar popular social media platforms. Facebook is a platform that tends to involve long messages and public conversations [46]. In contrast, Twitter is a platform for microblogging which allows only short messages fewer than 140 words until the end of 2017. Instagram is largely image-based rather than text-based. As social media platforms differ greatly in their characteristics, varying communication strategies may be adopted and required in different platforms. Future research should try to build a robust and full picture for strategic use of different platforms in the outbreak communication. Second, as the investigation is about Zika, which is an infectious disease that is new to the public, the strategies adopted by the government may differ from those for communicating a constant disease threat, such as dengue and influenza. Future research should compare the findings of this study to another similar disease outbreak. Third, the current research was conducted in Singapore, which is an internet-intensive country. Therefore, it is necessary to examine whether and how crisis communication on social media could work in a less internet-covered society. Past research has suggested that social media can act as an audience station to spread the information to those who are not inactive on social media [47]. Therefore, other than the direct crisis communication between governments and the public, the indirect communication between social media users and inactive users may be also essential. In this case, strategies should be adapted. Therefore, research with similar approach should be conducted in a less internet-intensive country.

Additionally, the findings and implications should be carefully examined when they are generalized to communicate other types of crises on social media. Some implications can be clearly generalized. For example, the utilization of social media for crisis education, and encouragement and regards of public cooperation can be replicated in different types of crises. Constant situation updates during a crisis to elicit discussions between the public and the government can also be a general principle [48]. In contrast, some other findings and implications may be specific to the outbreak context. For example, the importance of preparedness messages may not be generalized to some crises, such as natural disasters. It will be difficult to identify a pre-crisis stage for a particular disaster, such as an earthquake [45]. The preparation of a disaster crisis is likely done by long-term education on recommendations and self-efficacy promotions, rather than short-term preparedness via social media. Given that the CERC model is expected to be applied to all types of crises, it will be important to continually adapt and refine the CERC model for different crises contexts on social media.

## 5. Conclusions

Overall, this study is among the few to demonstrate how Facebook can be tactically utilized for outbreak communications by health authorities. The findings emphasize the importance of outbreak preparedness and strategic communications across various stages of the outbreak. This study provides important implications on an adapted CERC model within the context of social media. It also provides practical insights into the nuanced and novel use of social media to manage the outbreak.

## Figures and Tables

**Table 1:** Crisis and emergency risk communication model [5].

CERC Stage	Characteristics and Communication Aims
Pre-crisis	Risk messages, Warnings, Preparations; Communication and education campaigns targeted to both the public and the response community to facilitate:
	- Monitoring and recognition of emerging risks
	- General public understanding of risk - Public preparation for the possibility of an adverse event
	- Changes in behaviour to reduce the likelihood of harm (self-efficacy)
	- Specific warning messages regarding some eminent threat
	- Alliances and cooperation with agencies, organizations, and groups
	- Development of consensual recommendations by experts and first responders - Message development and testing for subsequent stages
Initial event	Uncertainty Reduction, Self-efficacy, Reassurance; Rapid communication to the general public and to affected groups seeking to establish:
	- Empathy, reassurance, and reduction in emotional turmoil - Designated crisis/agency spokespersons and formal channels and methods of communication
	- General and broad-based understanding of the crisis circumstances, consequences, and anticipated outcomes based on available information
	- Reduction of crisis-related uncertainty - Specific understanding of emergency management and medical community responses
	- Understanding of self-efficacy and personal response activities
Maintenance	Ongoing Uncertainty Reduction, Self-efficacy, Reassurance; Communication to the general public and to affected groups seeking to facilitate
	- More accurate public understandings of ongoing risks - Understanding of background factors and issues
	- Broad-based support and cooperation with response and recovery efforts - Feedback from affected publics and correction of any misunderstandings/rumors
	- Ongoing explanation and reiteration of self-efficacy and personal response activities - Informed decision making by the public based on understanding of risks/benefits
Resolution	Updates Regarding Resolution, Discussions about Cause and New Risks/New Understandings of Risk; Public communication and campaigns directed toward the general public and affected groups seeking to:
	- Inform and persuade about ongoing clean-up, remediation, recovery, and rebuilding efforts
	- Facilitate broad-based, honest, and open discussion and resolution of issues regarding cause, blame, responsibility, and adequacy of response.
	- Improve/create public understanding of new risks and new understandings of risk as well as new risk avoidance behaviours and response procedures
	- Promote the activities and capabilities of agencies and organizations to reinforce positive corporate identity and image
Evaluation	Discussions of Adequacy of Response; Consensus About Lessons and New Understandings of Risks; Communication directed toward agencies and the response community to:
	- Evaluate and assess responses, including communication effectiveness
	- Document, formalize, and communicate lessons learned
	- Determine specific actions to improve crisis communication and crisis response capability
	- Create linkages to precrisis activities

**Table 2:** Identified post categories.

Theme Category	Definition	Examples	Keywords
*Risk Messages*			
Disease mechanisms	Statements on disease mechanisms.	It is transmitted by the bite of infected Aedes mosquitoes (which bite in the daytime), identical to dengue.	Aedes, mosquito-borne
Symptoms	Statements on symptoms associated with Zika	Most infected persons may display mild or no symptoms.	symptom, mild, febrile, fever, rash*, *pain*, *ache*, conjunctivitis, microcephaly, redeye*, unwell
*Warnings*			
Risk factors	Statements with risk factors or risk groups associated with Zika	Pregnant travellers are advised to undertake strict precautions against mosquito bites.	pregnan*, travel*, sex, construction sites, mosquito bites, breeding, stagnant water
Danger	Statement that highlights the risk of Zika in Singapore	This is extremely critical and fundamental to our efforts to reduce the risk of further spread of the Zika virus.	risk, threat, concern*
*Preparations*			
Responders	Organizations or persons who will be responsible for the emergency	MOH and NEA were informed of the first case of locally transmitted Zika Virus Infection in Singapore.	NEA, MOH, The Ministry of Health, National Environment Agency
Recommendations	Requests and advises on taking actions to prevent Zika	We advise residents of Watten Estate, Casa Perla, Hillcrest Arcadia, The Arcadia and Watten Hill Condominium to monitor their health and seek medical attention if unwell.	advise, urge, please, recommend, precaution, take step, take action
*Uncertainty Reduction*			
Case report	reports and updates of case number and cluster	MOH and NEA were informed of the first case of locally transmitted Zika Virus Infection in Singapore.	case, cluster
Local locality	Statement on local geographic information of Zika	The patient is a 47-year-old female Malaysian who resides at Block 102 Aljunied Crescent and works in Singapore.	Aljunied Crescent, Sims Drive, Kallang Way, Paya Lebar Way, Bedok North, Joo Seng, Bishan, Woodland, Elite Terrace, Joo Avenue, Harvey Cresent, Siglap, Tagore, Ubi Cresent, Jalan Raya, Circuit Road, Sembawang Drive, Kranji Road, Senoko South Road, Lor 101 Changi, Toh Guan Road East, Joo Chiat Place, Watten Estate, Casa Perla, Hillcrest Arcadia, The Arcadia and Watten Hill, Geylang, area
Information resources	Websites or infographic that allow people to learn more about Zika	Read the press release here: https://www.moh.gov.sg/…/first-case-of-locally-transmitted-…	http, www., read, For more, health advisory, refer to, FAQ*, information, update*
*Efficacy*			
Personal prevention measures	Specific prevenetion actions one can take to prevent Zika	it is critical that all of us as a community take immediate steps to prevent mosquito breeding in our homes by doing the 5-step Mozzie Wipeout every alternate day.	repellent, screen*, condom, mozzie wipeout, mosquito nets, medical attention, prevent…by, protect…by, simple steps, we can, you can
Common responsibility	Exprssion of common responsibility for the public and other stakeholders.	Let’s prevent the spread of Zika in Singapore.	let’s, let us, all of us, each of us, everyone, all Singaporeans, work together, residents, we all
*Reassurance*			
Calming	Statements that remove uncertainty or fears of the Zika threat	NEA have introduced additional measures following the WHO’s declaration of an international public health emergency due to Zika’s link to the recent cluster of microcephaly cases in Brazil.	we will, we have, has/have been, NEA has, MOH has, MOH will, NEA will, NEA is, MOH is
Thanking and regards	Exprssion of thanks, approval and regards	I would like to thank residents, construction sites and dormitory operators for their co-operation, and urge them to continue to ensure that there is no breeding in their premises.	well done, thank*
Government interventions	Government intervention resposnes to Zika	MOH and NEA have since stepped up efforts to screen individuals and carry out vector control measures, so as to prevent the risk of further spread of the virus.	measure*, vector control, fog*, surveillance
*Dengue*			
Dengue	Mention of dengue	Together we can keep Zika and dengue at bay by dedicating just a few minutes of our day to doing the 5-step #MozzieWipeout	dengue
Wolbachia	Mention of Wolbachia	In the long term, we hope that the Wolbachia technology will eventually lead to a reduced urban Aedes aegypti mosquito population and hence, reduce the potential spread of diseases such as dengue and Zika.	Wolbachia

Note. Keywords indicates by * inlcude the derivatives. For example, measure* includes measure, measures, measured, measuring, and measurement, etc. Keywords were used for post categorization. If posts contain one of the keywords, they were categorized in that theme. The percentages listed are for the number in each group out of the total 72 posts.

**Table 3:** Post categories by crisis phases.

Theme Categories	Overall (*N* = 72)	Pre-Crisis (*N* = 11)	Initial Event (*N* = 54)	Maintenance (*N* = 7)	χ^2^
***Risk Messages***	***58 (81%)***	***10 (91%)***	***45 (83%)***	***3 (43%)***	7.37 *
Disease mechanisms	45 (63%)	8 (73%)	35 (65%)	2 (29%)	4.05
Symptoms	27 (38%)	7 (64%)	18 (33%)	2 (29%)	3.84
***Warnings***	***52 (72%)***	***10 (91%)***	***40 (74%)***	***2 (29%)***	8.66 *
Risk factors	49 (68%)	10 (91%)	37 (69%)	2 (29%)	7.67 *
Danger	19 (26%)	7 (64%)	12 (22%)	0 (0%)	10.85 **
***Preparations***	***65 (90%)***	***9 (82%)***	***53 (98%)***	***3 (43%)***	22.64 ***
Responders	53 (74%)	7 (64%)	44 (81%)	2 (29%)	9.60 **
Recommendations	40 (56%)	9 (82%)	28 (52%)	3 (43%)	3.83
***Uncertainty Reduction***	***63 (88%)***	***10 (91%)***	***50 (93%)***	***3 (43%)***	14.15 ***
Case report	52 (72%)	8 (73%)	42 (78%)	2 (29%)	7.48 *
Local locality	49 (68%)	5 (45%)	42 (78%)	2 (29%)	9.95 **
Information resources	56 (78%)	8 (73%)	45 (83%)	3 (43%)	6.07 *
***Efficacy***	***50 (69%)***	***9 (82%)***	***34 (63%)***	***7 (100%)***	***4.94***
Personal prevention measures	34 (47%)	8 (73%)	23 (43%)	3 (43%)	3.39
Common responsibility	45 (63%)	9 (82%)	30 (56%)	6 (86%)	4.47
***Reassurance***	***55 (76%)***	***7 (64%)***	***46 (85%)***	***2 (29%)***	12.18 **
Calming	52 (72%)	5 (45%)	45 (83%)	2 (29%)	13.90 ***
Thanking and regards	8.3 (6%)	1 (9%)	4 (7%)	1 (14%)	0.39
Government interventions	43 (60%)	7 (64%)	34 (63%)	2 (29%)	3.13
***Dengue***	***24 (33%)***	***4 (36%)***	***16 (30%)***	***4 (57%)***	***2.17***
Dengue	24 (33%)	4 (36%)	16 (30%)	4 (57%)	2.17
Wolbachia	2 (3%)	1 (9%)	1 (2%)	0 (0%)	2.00

Note. Percentages indicate the percentage of themed posts by all posts in a particular phase. Statistics indicate whether percentages significantly vary across phases for each of the theme. * *p* < 0.05; ** *p* < 0.01; *** *p* < 0.001.

**Table 4:** Public median engagement in themed posts.

Category	Likes				Shares				Comments			
	All	Pre	Outbreak	Post	All	Pre	Outbreak	Post	All	Pre	Outbreak	Post
All	17.5	43	17.5	8	0	26	1	0	2	2	2	0
Risk Messages	19	44.5	17	12	2.5	29	2	0	2	2.5	2	0
Warnings	18.5	39.5	13	13.5	1.5	23	1.5	0	2	2.5	2	0
Preparations	19	36	18	12	1	20	1	0	2	3	2	0
Uncertainty Reduction	19	44.5	17.5	12	2	29	1.5	0	2	2.5	2	0
Efficacy	22	43	22.5	8	0	26	0	0	2	3	2	0
Reassurance	19	46	17.5	13.5	3	26	2.5	0	2	3	2	0
Dengue	16.5	23	20.5	7.5	0	0	0	0	1	1.5	1.5	0

Note. Values are medians.

**Table 5:** Public median engagement in posts with vs. without topics in outbreak stages.

Phase	Variable	Engagement Variable	Median Present	Median Absent	*Z*	*p*
pre-outbreak	dengue	share	0	38	−2.71	0.007
outbreak	efficacy	like	22.5	13	2.72	0.007
	risk message	share	2	0	2.6	0.009
	uncertainty reduction	share	1.5	0	1.96	0.050
	uncertainty reduction	comment	2	0.5	2.55	0.011
	reassurance	share	2.5	0	2.9	0.004
	reassurance	comment	2	1	2.1	0.036

Note. Only significant results are presented.

**Table 6:** The adapted Crisis and Emergency Risk Communication Model (CERC) model in social media contexts based on current findings.

Stage	Communication Aims and Good Practices
Pre-crisis	Risk messages, Warnings, Preparations, Uncertainty reduction, Self-efficacy
	- Monitor and recognize emerging risks
	- Educate general public about risk - Warning regarding some eminent threat
	- Enhance awareness for the possibility of an adverse event
	- Design first responders and social media channels for communications *
	- Develop consensual recommendations by experts and first responders
	- Encourage public common responsibility *
	- Promote behavioural changes to reduce the likelihood of harm (self-efficacy)
	- *Reduce crisis-related uncertainty*
	- Establish official channels other than social media to provide further information *
	- Related healthcare education *
In-crisis	Ongoing Uncertainty Reduction, Self-efficacy, Reassurance; Discussions of Adequacy of Response
	- Reduce crisis-related uncertainty and emotional turmoil - Monitor and address instant feedbacks and concerns from the affected and general public *
	- *Facilitate understanding of emergency management and government interventions*
	- Promote self-efficacy and personal response activities
	- *Correct misunderstandings and rumours*
	- *Open discussion regarding blame, responsibility, and adequacy of response*
Post-crisis	Ongoing Uncertainty reduction, Self-efficacy; Regards, Educations
	- Provide continued information via official channels instead of social media updates *
	- Inform and persuade about ongoing clean-up, remediation, recovery, and rebuilding efforts
	- Ongoing encouragement of public common responsibility *
	- Ongoing promotion and reiteration of self-efficacy and personal response activities
	- Create linkages to future pre-crisis activities
	- Express regards about cooperation from other stakeholders *
	- Related healthcare educations *

Note. Practices followed by * indicate they are specific practices on social media; Practices being italic indicate they are practices of CERC model but may occur in a different phase.
